# Nationwide serological surveillance of non-tsetse-transmitted horse trypanosomoses in Mongolia

**DOI:** 10.1016/j.parepi.2020.e00158

**Published:** 2020-06-25

**Authors:** Daiki Mizushima, Tovuu Amgalanbaatar, Batdorj Davaasuren, Mitsunori Kayano, Sandagdorj Naransatsral, Punsantsogvoo Myagmarsuren, Davaajav Otgonsuren, Batsaikhan Enkhtaivan, Batbold Davkharbayar, Bayasgalan Mungun-Ochir, Purevdorj Baatarjargal, Uranbileg Nyamdolgor, Gurdorj Soyolmaa, Adilbish Altanchimeg, Myagmar Zoljargal, Thu-Thuy Nguyen, Badgar Battsetseg, Banzragch Battur, Noboru Inoue, Naoaki Yokoyama, Keisuke Suganuma

**Affiliations:** a National Research Center for Protozoan Diseases, Obihiro University of Agriculture and Veterinary Medicine, Inada, Obihiro, Hokkaido 080-8555, Japan; b Division of Medical Zoology, Department of Infection and Immunity, Jichi Medical University, School of Medicine, 3311-1 Yakushiji, Shimotsuke, Tochigi 329-0498, Japan; c Institute of Veterinary Medicine, Laboratory of Molecular Genetics, Mongolian University of Life Sciences, Zaisan 17024, Ulaanbaatar, Mongolia; d Research Center for Global Agromedicine, Obihiro University of Agriculture and Veterinary Medicine, Inada, Obihiro, Hokkaido 080-8555, Japan; e Obihiro University of Agriculture and Veterinary Medicine, Inada, Obihiro, Hokkaido 080-8555, Japan

**Keywords:** Castration, Dourine, ELISA, Horse trypanosomosis, Surra

## Abstract

In Mongolia, horses play important roles, not only in livestock production, but also in terms of culture, tradition, and Mongolian beliefs. Although the presence of non-tsetse-transmitted horse trypanosomoses, which are caused by infections with *Trypanosoma evansi* (surra) and *T. equiperdum* (dourine), has been reported in the country, whether there is a nationwide epidemic of these infectious diseases is unknown. In the present study, a nationwide surveillance of horse trypanosomoses was performed. The sample sizes for each province, the whole country, and male and female horses were, respectively, 96, 2,400, and 316 and 306. In total, 3,641 samples of horse sera were collected by simple random sampling. The rTeGM6-4r-based ELISA, which was applied for surra against cattle and water buffalo and dourine against horse, revealed that the overall sero-prevalence of the diseases in Mongolia was 4.8%. Among them, high sero-prevalences were observed in the central provinces (5.2–11.0%, *p* < 0.05) of the country. The sero-prevalence was significantly higher in females than in males (6.0% and 4.0%, *p* < 0.05, respectively) and in non-castrated males (8.4%, *p* < 0.01) compared with castrated males (3.0%). These results suggested that currently, horse trypanosomoses are a nationwide endemic problem in Mongolia. Knowledge of the nationwide endemic status of non-tsetse-transmitted horse trypanosomoses in Mongolia will be useful to prevent these diseases.

## 1 Introduction

The four million horses living in the whole area of Mongolia play important roles not only in livestock production (including transportation, milk, and meat production), but also in terms of culture, tradition, and Mongolian beliefs. It has been reported that non-tsetse-transmitted horse trypanosomoses, namely surra and dourine, were prevalent in Mongolia and the neighboring countries (Claes et al., 2005a; Clausen et al., 2003; Davaasuren et al., 2017; Luckins, 1988; Lun et al., 1992; OIE, 2013a; Suganuma et al., 2016). These reports raised concerns about the risk of these diseases spreading and suggested that their spread would cause huge economic losses in terms of horse production. Nevertheless, because there is no nationwide epidemiological information on these diseases, an effective approach to their control has not yet been initiated.

*Trypanosoma evansi* and *T. equiperdum* are the respective etiological agents of surra and dourine. *T. evansi* has a huge host range and is widely distributed throughout the world (Brun et al., 1998). *T. evansi* is mechanically transmitted by biting flies such as tabanids and *Stomoxys* (Desquesnes et al., 2013; Baldacchino et al., 2014). The frequent blood sucking by these flies may be the cause of infection between herds in the same place (Desquesnes et al., 2013). Moreover, transmission may occur by vertical, horizontal, iatrogenic, and oral means (Raina et al., 1985; Ogwu and Nuru, 1981; Desquesnes et al., 2013). The infection causes fetal diseases such as fever, anemia, and edema, especially in horse and camel (Desquesnes et al., 2013). *T. equiperdum* is also widely distributed in the world. This parasite specifically infects *Equidae* via coitus and causes a chronic and/or acute disease (Claes et al., 2005b; Gizaw et al., 2017). *T. equiperdum* is associated with the characteristic features of the parasite, which primarily parasitizes in the genital mucosa of equids and rarely parasitizes in blood, dissimilar to *T. evansi* (Brun et al., 1998). This disease is characterized by genital lesions, cutaneous plaques, and nervous signs, which are similar to those of surra (Claes et al., 2005b; Gizaw et al., 2017).

The World Organisation for Animal Health (OIE)-recommended serological tests, the CATT (card agglutination test for trypanosomosis) and a crude antigen-based enzyme-linked immunosorbent assay (ELISA), rely on the preparation of trypanosome antigens (OIE, 2017). In particular, cross-reactions with *Theileria* spp., *Babesia* spp., and *T. theileri* in water buffalo were reported with the crude antigen-based ELISA (Nguyen et al., 2014). Other diagnostic formats for trypanosomoses are the indirect fluorescent assay and complement fixation test. However, it has recently been reported that it is difficult to distinguish *T. evansi* and *T. equiperdum* with these diagnostic techniques (Claes et al., 2005b; OIE, 2013b). In contrast, several serological tests have been developed using recombinant tandem repeat proteins, which are often targeted for B-cell responses (Nguyen et al., 2014; Goto et al., 2007; Nguyen et al., 2015). Among these proteins, the GM6 protein was found to be a highly reactive antigen that can be used for the diagnosis of animal trypanosomosis (Nguyen et al., 2014; Nguyen et al., 2015; Nguyen et al., 2012). Since the discovery of GM6 as a diagnostic antigen for the detection of animal trypanosomosis, it has received scientific attention with the application of rTeGM6-4r (recombinant *T. evansi* GM6 4-repeat), which is derived from *T. evansi* GM6-4r antigen, to develop an ELISA and immunochromatographic test for sero-surveillance of surra in cattle and water buffalo (Nguyen et al., 2014; Nguyen et al., 2015). Compared with *T. evansi* crude antigen ELISA, the rTeGM6-4r antigen-based ELISA shows little cross-reactivity for *Theileria* spp. and *Babesia* spp., which are etiological parasites for equine piroplasmosis and are found in Mongolian horses (Munkhjargal et al., 2013; Nguyen et al., 2014). Furthermore, the rTvGM6 derived from *T. vivax* was developed as a diagnostic antigen for the point-of-care diagnosis of disease caused by *T. vivax* (Boulangé et al., 2017; Pillay et al., 2013). Our previous study also indicated that the rTeGM6-4r-based ELISA and the immunochromatographic test are suitable tools in the sero-surveillance of non-tsetse-transmitted horse trypanosomoses (Davaasuren et al., 2017; Mizushima et al., 2018). The aim of the present study was to assess the epidemic situation of non-tsetse-transmitted horse trypanosomoses as a basis for formulating an effective approach to control these diseases in Mongolia.

## 2 Materials and methods

### 2.1 Horse serum samples in Mongolia

In total, 3,641 samples of horse sera were collected by simple random sampling from horses with information on age groups according to first reproduction age (1–4 years old =1,337; 5 years and older = 1,922) (Hund, 2008), sex (*n* = 3,267), and castration status (*n* = 541) that had been maintained in all 19 Mongolian provinces from July 2014 to December 2017. The sample sizes for each province, the whole country, and male and female horses were, respectively, 96, 2,400, and 316 and 306. The sample sizes were calculated according to the number of living horses in each province, which was recorded in 2017 (NSOM, 2017), by an OpenEpi program setting under the parameters of confidence level 95%, confidence limit 10%, and anticipated frequency 50% (http://www.openepi.com/Menu/OE_Menu.htm). However, the minimum sample size of 96 was not achieved in some provinces (Arkhangai: 82, Dornogovi: 94, Govisumber: 69).

The blood samples were collected from the jugular vein of the horses. Serum samples were prepared from these horses' blood samples and were stored at −30 °C until use. ELISA was applied to all serum samples in Japan as described in Section 2.2. Based on geographical factors, some small provinces were enclosed in neighboring large provinces; thus, the serum samples collected in Ulaanbaatar, Darkhan-Uul, and Orkhon provinces were combined with the samples from Tov, Selenge, and Bulgan provinces, respectively.

Five positive and 19 negative reference sera were prepared from horses in Mongolia that had been diagnosed as positive and negative, respectively, by clinical evidence, polymerase chain reaction, CATT, and microscopic examination of trypanosomes (Davaasuren et al., 2017; Mizushima et al., 2018; Suganuma et al., 2016). One of each of these positive and negative reference sera was consecutively used for ELISA as the positive and negative controls in this study.

### 2.2 ELISA

ELISA was performed following a previously described procedure with minor modifications (Davaasuren et al., 2017). Briefly, an rTeGM6-4r antigen was prepared according to the procedure in our previous study (Nguyen et al., 2015). Each well of an ELISA plate (Thermo Fisher Scientific, Waltham, MA) was first coated with 100 μL of 2 μg/mL rTeGM6-4r antigen in carbonate buffer. Subsequently, 350 μL of phosphate-buffered saline with 0.05% Tween 20 (PBS-T) containing 3% skim milk was applied to each well. After blocking at 37 °C for 2 h, horse serum samples diluted 200 times were applied to each well and then incubated at 37 °C for 2 h. After washing with PBS-T, horseradish peroxidase-conjugated anti-horse IgG-heavy- and light-chain goat antibodies (Bethyl Laboratories, Inc., Montgomery, AL) diluted 40,000 times in PBS-T with 3% skim milk were applied to each well. The ELISA plates were incubated at 37 °C for 1 h and then washed with PBS-T. Finally, a TMB (3, 3′, 5, 5′-tetramethylbenzidine) peroxidase substrate system (SeraCare Life Sciences, Milford, MA) was used to detect the antigen-antibody reaction. The absorbance of each well was measured at 450 nm in a GloMax®-Multi+ Detection System (Promega, Madison, WI). The cut-off values, based on the three-sigma rule in statistics, were calculated from the mean of the optical density values of negative reference sera samples plus 3-times standard deviation (Lardeux et al., 2016). These analyses were performed in duplicate.

### 2.3 Statistical analysis

Based on the results of the rTeGM6-4r-based ELISA, sero-prevalences were calculated by using an OpenEpi program (http://www.openepi.com/Menu/OE_Menu.htm). Statistical analyses were performed with the Chi-squared test by using GraphPad Prism version 6 (GraphPad Software, La Jolla, CA). Logistic regression was applied to the same data set with province and sex as explanatory variables using R version 3.1.2 (R Foundation for Statistical Computing, 2014).

## 3 Results

**Fig. 1:**
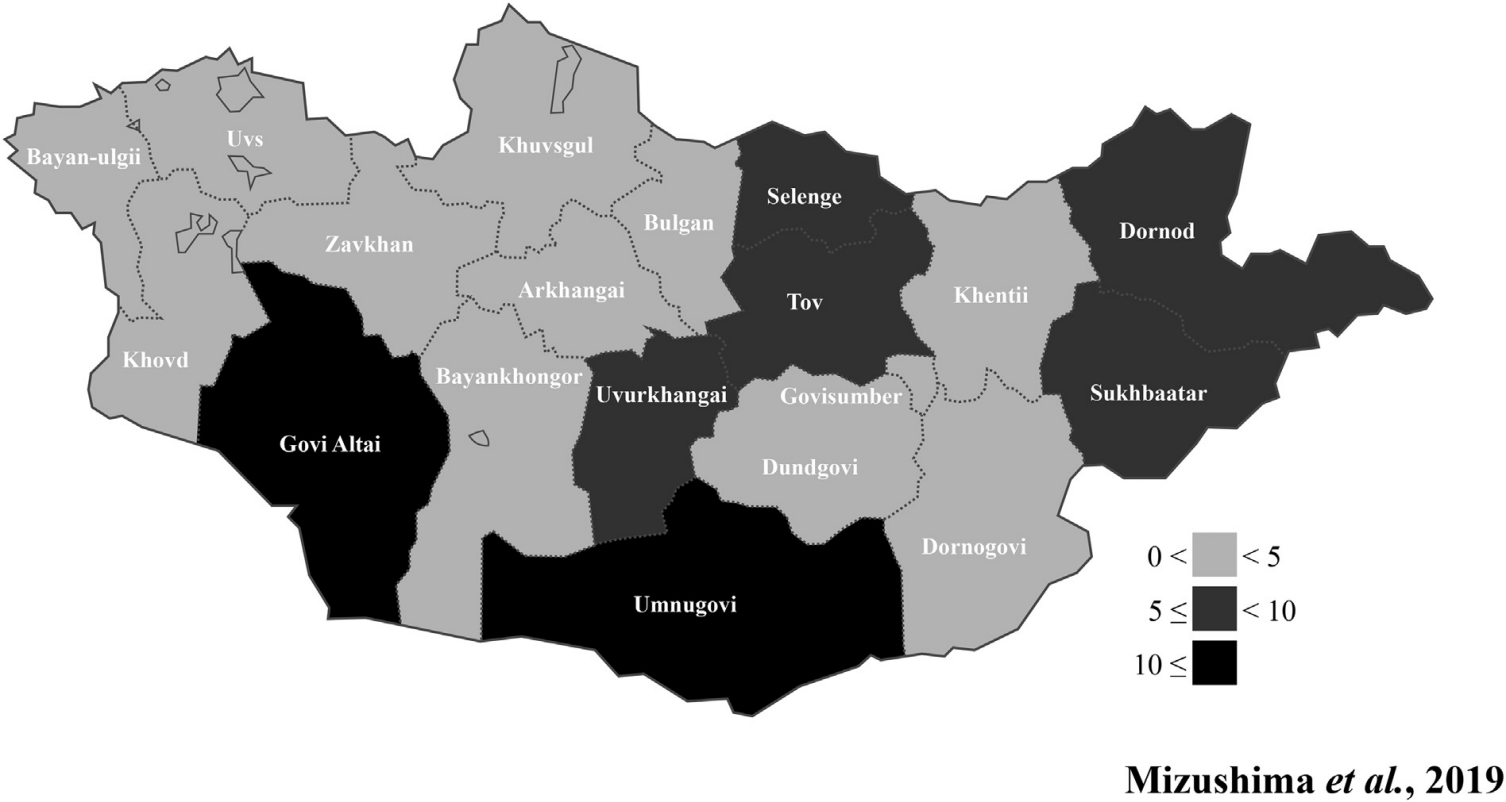
Epidemiological mapping of non-tsetse-transmitted horse trypanosomoses. Each province is distinguished by a color gradation, which depends on the sero-prevalences for non-tsetse-transmitted horse trypanosomes. The ranges of the positive ratio for each color gradation are indicated in the figure. The small provinces enclosed in Tov, Selenge and Bulgan provinces are Ulaanbaatar, Darkhan-Uul, and Orkhon provinces, respectively.

Among the 3,641 serum samples, 173 were sero-positive for non-tsetse-transmitted horse trypanosomes in the rTeGM6-4r-based ELISA. Sero-positive samples were found in each province (Fig. 1). The overall sero-prevalence was 4.8% (Table 1). The highest sero-prevalences as indicated by Chi-square test were observed in Govi-Altai (10.4%, *p* < 0.01) and Umnugovi (11.0%, *p* < 0.01) provinces (Table 1). Moreover, high sero-prevalences were observed in Dornod (5.2%), Uvurkhangai (5.9%), Selenge (6.6%, *p* < 0.1), Sukhbaatar (7.0%, *p* < 0.05), and Tov (8.5%, *p* < 0.01) provinces (Table 1, Fig. 1). The lowest sero-prevalences were observed in the other provinces (0.6%–4.6%). There was no significant difference in sero-prevalence between horses aged 1–4 years old (4.3%) and those 5 years or older (5.3%) (Table 2). Of the 1,774 male serum samples, 71 were sero-positive, whereas 89 of the 1,493 female serum samples were sero-positive. The sero-prevalence of the female horses (6.0%, *p* < 0.01) was significantly higher than that of the male horses (4.0%) (Table 2). Moreover, the sero-prevalence in the uncastrated males (8.4%) was significantly higher than that in the castrated males (3.0%, *p* < 0.01)(Table 2). These results indicated that province, sex, and castration status were related to sero-prevalence.

**Table 1:** Sero-prevalences for non-tsetse-transmitted horse trypanosomoses in different provinces.

Province	Serum samples	Positive sera	Positive ratio (%)	95% Confidence interval
Arkhangai	82	1	1.2	0.2–6.6
Bayan-Ulgii	280	5	1.8	0.8–4.1
Bayankhongor	134	6	4.5	2.1–9.4
Bulgan	180	3	1.7	0.6–4.8
Dornod	194	10	5.2	2.8–9.2
Dornogovi	94	4	4.3	1.7–10.4
Dundgovi	164	1	0.6	0.1–3.4
Gobi Altai^b^, ^C^	96	10	10.4	5.8–18.1
Govisumber	69	3	4.3	1.5–12.0
Khentii	194	9	4.6	2.5–8.6
Khovd	225	5	2.2	1.0–5.1
Khovsgol	236	4	1.7	0.7–4.3
Selenge^A^	151	10	6.6	3.6–11.8
Sukhbaatar^a^, ^B^	330	23	7.0	4.7–10.2
Tov^b^, ^C^	434	37	8.5	6.2–11.5
Umnugovi^b^, ^C^	155	17	11.0	7.0–16.9
Uvs	136	4	2.9	1.2–7.3
Uvurkhangai	202	12	5.9	3.4–10.1
Zavkhan	285	9	3.2	1.7–5.9
Total	3,641	173	4.8	4.1–5.5

a *p* < 0.05, Chi-square test.

b *p* < 0.01.

A *p* < 0.1, Logistic regression with provinces as explanatory variables.

B *p* < 0.05.

C *p* < 0.01.

**Table 2:** Sero-prevalences for non-tsetse-transmitted horse trypanosomoses by age groups, sex and castration status.

Items	Serum samples	Positive sera	Positive ratio (%)	95% Confidence interval
Age groups				
1–4	1,337	58	4.3	3.4–5.6
≥5	1,922	102	5.3	4.4–6.4
Total	3,259	160	4.9	4.2–5.7
Sex^A^				
Male	1,774	71	4.0	3.2–5.0
Female^a^	1,493	89	6.0	4.9–7.3
Total	3,267	160	4.9	4.2–5.7
Castration				
Castrated	363	11	3.0	1.7–5.3
Uncastrated^a^	178	15	8.4	5.2–13.4
Total	541	26	4.8	3.3–6.9

a *p* < 0.01, Chi-square test.

A *p* < 0.05, Logistic regression with sex and castration as explanatory variables.

Logistic regression was carried out on the dataset with provinces and sex as the explanatory variables. The analysis showed both variables to be significantly associated with sero-prevalence, whereas the analysis including castration as an explanatory variable showed it not to be associated with sero-prevalence (*p* = 0.13) (Table 2). The logistic regression analysis estimated province and sex to be the factors related to the increase in sero-prevalence.

## 4 Discussion

Several reports have indicated the risk of an epidemic of non-tsetse-transmitted horse trypanosomoses in Mongolia (Clausen et al., 2003; Davaasuren et al., 2017; Suganuma et al., 2016). Therefore, it is important to assess the epidemic status of these infectious diseases nationwide to plan for their prevention and control. In the present study, we revealed the nationwide endemic status of horse trypanosomoses in Mongolia.

In countries free of horse trypanosomoses, horses suspected of having dourine are euthanized according to the stamping-out policy recommended by the OIE (OIE, 2017). However, it is difficult to euthanize horses in Mongolia because of their importance in the traditions and beliefs of the Mongolians. There are several reports of diminazene diaceturate (Diminasan®) and bis (aminoethylthio) 4-melaminophenylarsine dihydrochloride (Cymelarsan®) having chemotherapeutic effects on the two horse trypanosomoses (Hagos et al., 2010; Tuntasuvan et al., 2003). In our recently published study, combination chemotherapy improved the clinical symptoms of dourine in Mongolia (Davkharbayar et al., 2020). We have developed the rTeGM6-4r-immunochromatographic test (rTeGM6-4r-ICT), which is a field-friendly, rapid diagnostic tool (Mizushima et al., 2018). With it, the rural area veterinarian can diagnose the diseases without special equipment. Therefore, to prevent further spreading of the disease, screening by rTeGM6-4r-ICT and administration of chemotherapy in sero-positive horses including those exhibiting clinical symptoms needs to be started, especially in Govi-Altai, Selenge, Sukhbaatar, Tov, and Dornod provinces, in which the sero-prevalences of horse trypanosomoses are significantly high.

Reports on livestock migration in Mongolia have indicated that horses are concentrated in the central areas of Tov and surrounding provinces (Janzen, 2005; Saizen et al., 2010). Currently, cross-breeding between Mongolian horses and imported horses is frequently conducted in Mongolia, especially in Tov province, because relatively high numbers of high-income earners are living in the area. However, due to insufficient quarantine services, imported horses with trypanosomoses were not quarantined. Such a high concentration of horses and mating with non-quarantined imported horses in this area might have induced the relatively high sero-prevalences found in Tov and the surrounding provinces. Unfortunately, the reason for high prevalences in other provinces being veiled is due to the lack of official information on reproduction and migration.

We found no relationship between age group and sero-prevalence although it was estimated that the manner in which dourine spreads was related to age groups as first reproduction is usually carried out at 5 years old (Hund, 2008). There is a non-official report in Mongolia that camels suspected of having surra (*T. evansi* infection) were previously found in the western part of Mongolia. If the epidemic of surra was caused by the mechanical transmission of biting flies, there might be no significant differences in sex or castration status due to unselective blood feeding by the biting flies. It is likely that the causes of infection in the 1–4-year-old horses might be the vertical transmission of either or both surra and dourine and the mechanical transmission of surra by biting flies. However, we recently reported a clinical case of a dourine-endemic farm in which we successfully isolated *T. equiperdum* from infected horses and parasitologically confirmed the clinical cases as dourine (Davaasuren et al., 2017; Suganuma et al., 2016; Mungun-Ochir et al., 2019). The present study indicated a high prevalence of horses capable of mating and of females and uncastrated males (Table 2), and it appeared that the infection of these horses with dourine might have been caused by coitus (Calistri et al., 2013). However, our results could not distinguish between *T. evansi* and *T. equiperdum* due to limitations of sero-diagnosis. These findings have encouraged us to further investigate the details of the mechanism of spread in Mongolia, perform research into the ecology of biting flies and vertical and sexual transmission through experimental infection in horse, and develop a means of definitively diagnosing these two diseases.

Sero-prevalence in the present study appeared to be related to sex and the castration status of the male horses. However, it is unclear whether castration status is related to the sero-prevalence of each province because the lack of an association between castration status and sero-prevalence might be related to the few positives recorded for each province in the dataset containing castration information (*n* = 541) (Table 2).

To monitor the occurrence of disease in Mongolia, nationwide surveillance and an attempt to isolate the trypanosomes will need to be carried out continuously. Recently, we completed whole genome sequencing of *T. equiperdum* IVM-t1, Mongolian strain (Davaasuren et al., 2019). In the near future, it is hoped that *T. equiperdum*-specific genes will be found to improve the tools to serologically distinguish between *T. evansi* and *T. equiperdum*. In conclusion, the present study was the first report, to our knowledge, to show the nationwide endemic status of non-tsetse-transmitted horse trypanosomoses in Mongolia.

## Declaration of Competing Interest

The authors declare no competing interests in association with this study.
